# Identifying Genes that Help One Sex but Harm the Other

**DOI:** 10.1371/journal.pbio.1000336

**Published:** 2010-03-16

**Authors:** Robin Meadows

**Affiliations:** Freelance Science Writer, Fairfield, California, United States of America

Traits that help one sex can harm the other, resulting in conflicting evolutionary pressures on males and females. In zebra finches (*Taeniopygia guttata*), for instance, males with redder bills attract more mates but females with redder bills attract fewer mates. This battle of the sexes is thought to extend to the genetic level, with individual genes favoring one sex over the other. Some of the strongest evidence for these sexually antagonistic genes comes from studies showing that fruit fly lines with high reproductive success in one sex typically have low reproductive success in the other. Thus, if males in a particular line have many offspring, the females do not and vice versa.

The genes underlying this sexual tug-of-war, however, have been difficult to find. Now, Paolo Innocenti and Edward Morrow reveal this conflict's genetic basis by linking the expression of sexually antagonistic genes in the fruit fly *Drosophila melanogaster* to the reproductive success of one sex at the expense of the other.

The first step was identifying fruit fly lines where males and females had differential reproductive success or fitness. The researchers tested the fitness of males and females in 100 lines of fruit flies. For each line, test males and competitor males vied for mates. The test males had the normal red eyes, whereas the competitor males and females had brown eyes. Because red eyes are dominant, the proportion of red-eyed offspring yielded the relative fitness of males in each line. Similarly, females from each line and competitor females vied for food, with the number of offspring yielding the relative fitness of females in each line. As expected, lines with high-fitness males generally had low-fitness females, whereas lines with high-fitness females generally had low-fitness males.

Next, Innocenti and Morrow compared gene expression in five lines with high male and low female fitness, five lines where the reverse held, and five lines where the fitness of the two sexes was average. RNA analysis of the males and females in these 15 lines revealed that although most gene expression was sex-biased, only a minority of transcripts were consistently associated with male or female fitness. While about 17,000 transcripts, or about 90% of the total, were expressed at higher levels in one sex or the other, only 867 were associated with male fitness and only 634 were associated with female fitness.

To identify which genes were sexually antagonistic, the researchers determined which of these transcripts were associated with fitness in one sex at the expense of the other. They found that nearly 1,500 transcripts benefitted one sex over the other, with roughly half favoring males and half favoring females. These transcripts correspond to nearly 1,300 known genes, and these sexually antagonistic genes comprise the majority (about two-thirds) of the sex-specific fitness genes. This suggests that sexually antagonistic selection maintains genetic variation for fitness.

The researchers were surprised that sexually antagonistic genes accounted for so few of the sex-biased transcripts, which were expressed at higher levels in one sex over the other, but were not necessarily associated with fitness. Sex-biased gene expression has been used as a proxy for sexual antagonism, based on the assumption that these differences in expression reflect conflict at the genetic level. However, this study questions that approach: sexually antagonistic genes accounted for a mere 8% of the total sex-biased transcripts.

Innocenti and Morrow also tested—and confirmed—the key prediction that sexually antagonistic genes should accumulate on sex chromosomes. Because these chromosomes are inherited differently by males and females, this should help resolve the evolutionary conflict between the sexes. X chromosomes could have a recessive “male fitness” gene, for instance, which would benefit males with that gene on their single X chromosome. But this male benefit would not necessarily come at the expense of females: they would not be harmed if this recessive gene was on only one of their X chromosomes. As expected, the researchers found that sexually antagonistic genes were overwhelmingly concentrated on the X chromosome.

Previous studies suggest that sexually antagonistic genes are widespread amongst animals, from insects to birds to mammals. By offering this initial glimpse of the genetic basis of such findings, Innocenti and Morrow lay the groundwork for pinpointing the locations and functions of these genes that pit males and females against each other.

**Innocenti P, Morrow EH (2010) The Sexually Antagonistic Genes of** ***Drosophila melanogaster*** **. doi:10.1371/journal.pbio.1000335**

**Figure:**
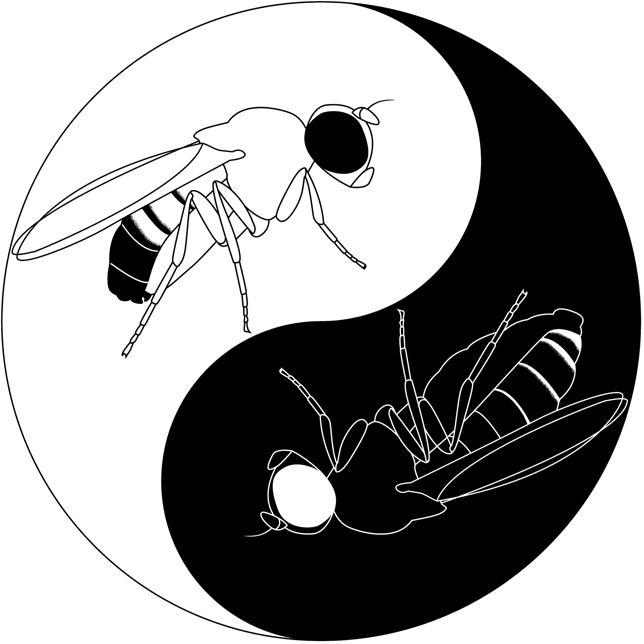
Males and females display the yin-yang duality represented in the Taijitu (above): opposing forces (sexually antagonistic selection) tend to drive them apart, but an ontogenic constraint—the shared genetic material—prevents their disjunction. Image: Ika Österblad.

